# Infants Younger Than 90 Days Admitted for Late-Onset Sepsis Display a Reduced Abundance of Regulatory T Cells

**DOI:** 10.3389/fimmu.2021.666447

**Published:** 2021-08-27

**Authors:** Ingmar Fortmann, Marie-Theres Dammann, Bastian Siller, Alexander Humberg, Martin Demmert, Ludger Tüshaus, Judith Lindert, Vera van Zandbergen, Julia Pagel, Jan Rupp, Egbert Herting, Christoph Härtel

**Affiliations:** ^1^Department of Pediatrics, University of Lübeck, Lübeck, Germany; ^2^German Center for Infection Research (DZIF), Partner Site Hamburg-Lübeck-Borstel-Riems, Lübeck, Germany; ^3^Department of Pediatric Surgery, University of Lübeck, Lübeck, Germany; ^4^Department of Infectious Diseases and Microbiology, University of Lübeck, Lübeck, Germany; ^5^Department of Pediatrics, University Hospital of Würzburg, Würzburg, Germany

**Keywords:** neonatal immunity, sepsis, infants < 90 days, regulatory T cells, invasive bacterial infection, lymphocyte subsets, sepsis workup

## Abstract

**Objective:**

To provide epidemiological data of infants < 90 days of age with suspected late-onset sepsis (LOS) and evaluate distinct immunological specificities. We hypothesized that previously healthy infants < 3 months of age with sepsis have a yet undefined immunological predisposition; e.g. differences in lymphocyte subsets including regulatory T cells.

**Methods:**

We performed an exploratory, single center study between January 1^st^, 2019 and June 1^st^, 2021. Routine diagnostics included conventional culture (blood, cerebrospinal fluid, urine), PCR and inflammatory markers in infants < 90 days of age with suspected sepsis. We additionally analyzed lymphocyte subsets and CD4+ CD25+ forkhead box protein (FoxP3)^+^ Tregs at admission for sepsis workup as compared to age-matched controls.

**Results:**

A convenience sample cohort of n= 51 infants with sepsis workup was enrolled. Invasive bacterial infection (IBI) was diagnosed in 25 (49.0%) patients including two infants with a rhinovirus co-infection and viral infection in 14 (27.5%) neonates. No infectious cause was found in 12 cases. Infants with suspected LOS displayed a decreased abundance of CD4+ FoxP3+ T cells as compared to controls, which was most pronounced in the subgroup of infants with IBI. We also noticed elevated HLA-DR-positive CD3+ cells in infants with LOS and a higher CD4/CD8-ratio in infants with viral infection as compared to healthy controls. Infants with viral infections had a higher number of natural killer cells as compared to infants with IBI.

**Conclusion:**

Our exploratory data support the concept of a potential immaturity state and failed immune tolerance development for young infants with LOS. Future large-scale studies are needed to elucidate pre-sepsis conditions and to target the microbiome-immunity interplay as a potential risk pattern.

## Introduction

Episodes with suspected infections are highly relevant in early infancy, particularly in the first three months of life (1). Late-onset sepsis (LOS) in infants < 90 days of age presents with non-specific clinical signs such as fever. Diagnostic approaches are imprecise due to low sensitivity of cultures and limited specificity for laboratory markers, e.g. C-reactive protein or procalcitonin (2). As infections in young infants often have a fulminant course, antibiotic treatment is consequently initiated as soon as clinical suspicion is evident (3–5). However, diagnostic markers or signatures to clearly differentiate between invasive bacterial infection (IBI), viral infection or no infection are lacking. Molecular biology tools (e.g. multiplex pathogen-PCR; next-generation sequencing) are cost-intensive and not available as specific bedside tests to guide a medical decision for antibiotic treatment (5). There are previous studies indicating that 7-13% of infants < 90 days of age have invasive bacterial infection (IBI; including urinary tract infection, UTI; bacteremia and/or meningitis) (3, 6) but often causes of fever remain unknown. The diagnosis of a viral infection may help to discontinue antibiotic therapies and herewith reduce the total exposure in these infants (7).

From an immunological perspective, the group of infants admitted with suspected LOS < 90 days of age has largely been neglected for comprehensive research approaches. We herein address the hypothesis of a potential immaturity state and failed immune tolerance development for young infants with suspected LOS. Historically, neonatal T cells – when compared to those from adults - were considered immature, less functional or unresponsive against specific antigens. This idea has been recently replaced by a concept including the important role of regulatory T cells in the immunological adaptation to the rapidly changing environment in the first months of life (8, 9). Neonatal T cells, in particular CD4+ and CD8+ cells, combine functionality in protecting from foreign pathogen as well as sustaining tolerance to self-antigens. Regulatory T cells (Tregs) represent a subpopulation of CD4+ T cells with an ability to limit the immune response against self- and non-self-antigens. This temporary feature seems to be crucial for the ontogenetic control of immune activation and feto-maternal tolerance. An increased abundance of Tregs in preterm infants, however, correlates with sepsis risk, possibly through their immunosuppressive capacity (10). In term infants, Tregs have been shown to be less abundant in peripheral blood during *RSV* infection (11, 12). Further, Tregs are reduced in infants prone to allergies (13) whereas in adults Tregs are upregulated in the course of sepsis which correlates to mortality risk (14). B cells inherit an important role in adult sepsis - patients’ immunological responses in an antibody-dependent and antibody-independent manner (15). In neonates, B cells lack antigenic exposure and have limited expression of B cell receptors resulting in low levels of primary IgG responses to immunizations and infections (16). However, the role of B cells and NK cells in the neonatal response to sepsis is incompletely understood and mainly studied in preterm infant cohorts (17, 18). In animal models, activation of NK cells has been shown to alter neonatal sepsis outcomes, hence they are proposed as candidate for immunomodulatory prophylactic treatment (19). Reduced abundances of NK cells at birth have proved to be associated with increased risk for LOS in human term and preterm neonates (18).

The distinct immune responses of the newborn mediate the transition from an intrauterine environment to an extrauterine life. Age-dependent susceptibility to infection remains an important threat in young infancy and reflects developmental trajectories in cell autonomous immunity (CAI), nutritional immunity and metabolic pathways such as serum-iron concentrations, postnatal development of physiological barriers and production of antimicrobial proteins and peptides (APP) (9).

The scope of the present study was to delineate whether the group of infants < 90 days with an uneventful postnatal period and suspected LOS carries certain immunological characteristics that are related to lymphocyte subsets or Treg frequencies.

## Materials and Methods

Between January 1^st^, 2019 and June 1^st^, 2021, we performed a convenience sample, single-centre observational study (Fever Without Source study; FWS study) with infants that were admitted from the community for suspected sepsis with an uneventful previous history for infections. The inclusion criteria were age < 90 days and > 72 hours and suspected late-onset sepsis with sepsis workup and empirical antibiotic treatment. Afebrile infants admitted to hospital for non-infectious reasons (i.e. with elective surgery) in the first 3 months of life served as controls. After written informed consent was given by the parents, infants with suspected LOS and controls were enrolled by the attending physicians.

Besides routine sepsis diagnostics (“sepsis work-up”: biomarkers of inflammation, conventional cultures of blood, urine and cerebrospinal fluid), we performed extended viral diagnostics including an in-house multiplex PCR from nasopharyngeal aspirates (NPA) and enterovirus in-house PCR from stool samples at the local Institute for Clinical Chemistry. In infants with suspected meningitis the enterovirus PCR from stool samples was performed by the institute for hygiene and environment in Hamburg (surveillance program for enterovirus meningitis/encephalitis). The multiplex-assay from NPA (*RP2Plus Biofire* ^®^ *respiratory 2.1 plus panel*) included *adenovirus, human rhino-/enterovirus, respiratory syncytial virus (RSV), influenza virus A and B, parainfluenza virus 1–4, metapneumovirus, coronavirus V (4 subtypes: HKU1, NL63, 229E, OC43), MERS CoV, Bordetella pertussis and parapertussis, chlamydia pneumoniae, mycoplasma pneumoniae*. Stool samples were tested for *rotavirus, adenovirus, and norovirus* using commercially available antigen tests (Ridascreen, r-Biopharm, Darmstadt, Germany), when infants developed symptoms of gastroenteritis.

The infants’ clinical and demographic characteristics, laboratory markers, and clinical presentation were immediately documented in a written case report form. Following pseudonymization, study personnel transferred the data into a prespecified Microsoft Excel database (Microsoft Office 2010, Versions 14.0). Data of all infants were monitored by two researchers (IF and MTD) using the original patient files. The diagnostic results were not blinded to the clinicians in the unit.

### Ethics

All study parts were approved by the local committee on research in human subjects at the University of Lübeck (Reference number: 20-228). Blood samples were obtained exclusively within a medically required blood withdrawal at admission and before any treatment with anti-infective agents. The additional blood volume (< 1% of body blood volume per blood sampling) was in line with current guidelines of the European Medical Agency (EMA) on the investigation of medicinal products in term and preterm infants; Committee for Medicinal Products for Human Use and Pediatric Committee (PDCO, 2009).

### Definitions

**Gestational age** was calculated from the best obstetric estimate based on early prenatal ultrasound and obstetric examination. **Late-onset sepsis** (LOS) was defined as sepsis occurring after the first 72 h of life. **Clinical LOS** was defined as pediatricians decision to treat the infant with antibiotics for at least 5 days due to the following reasons: two clinical signs of systemic inflammatory response: temperature > 38.0°C or < 36.5°C, tachycardia > 200/min, new-onset or increased frequency of bradycardias or apneas, hyperglycemia > 140 mg/dl, base excess < 210 mval/l, changed skin color and increased oxygen need; and one laboratory sign: C-reactive protein > 10 mg/l, platelet count < 100/nl, immature/total neutrophil ratio>0.2 and white blood cell count<5/nl (20). **Culture-confirmed sepsis** was defined as clinical sepsis with proof of causative agent in cultures of blood, urine or cerebrospinal fluid. **Invasive bacterial infection** (IBI) was defined as culture-confirmed sepsis or clinical sepsis. **Viral infection** was defined as proof of causative virus *via* multiplex PCR from nasopharyngeal aspirate, enterovirus PCR from stool samples, *RSV* rapid test, influenza rapid test or PCR test for *adenovirus, norovirus* and *rotavirus*. **Fever** was defined as central body temperature of > 38.0°C.

### Analysis of White Blood Cell Counts, Lymphocyte Subsets and Tregs

After blood withdrawal, the EDTA whole blood samples were stored for a maximum of 24 hours at room temperature before processing (21). As part of the clinical routine laboratory evaluation, white blood cell counts including numbers of lymphocytes were assessed by the central laboratory of the university hospital from EDTA blood (ethylenediamine tetraacetic acid). Further immunological analyses were performed in two different laboratories: First, the central laboratory of the university hospital performed the determination of lymphocyte subsets (CD3+/CD4+/CD8+/CD19+/[CD16/CD56]+/activated T cells and the CD4/CD8-ratio) on weekdays, Monday–Friday from 8a.m. to 4p.m. Counting of lymphocyte subsets and their activation status in whole blood was performed on a BD FACSCanto ™ II system (BD Biosciences, San Jose, CA, USA) with BD FACSCanto clinical software using multitest 6-Color TBNK (T cells, B cells and natural killer cells) kits and multitest CD3/CD8/CD38/HLA-DR kits according to the manufacturer’s instructions. Cytometer performance was checked weekly using BD FACS 7-Color Setup Beads, while the quality controls BD Multi-Check Control and BD Multi-Check CD4 Low Control were run alternately twice a day. Gating strategies and representative plots are available in the BD user manual *BD Multitest™ 6-color TBNK* (22).

Second, we performed immunological analyses in the research laboratory of the pediatric department as previously described (10, 23). The analyses included the determination of CD3+ lymphocytes, CD4+ lymphocytes, CD3+ CD4+ lymphocytes, CD3+ CD4+ CD25+ lymphocytes, CD3+ CD4+ Foxp3+ lymphocytes and CD3+ CD4+ Foxp3+ CD25+ lymphocytes. A cell viability test was performed on a regular basis to control for dead cells (eBioscience, San Diego, CA, USA). We used flow cytometry to determine the cell population percentages and absolute cell counts (**Supplementary Table 1**). In order to characterize T cell populations, we performed a whole blood staining with fluorochrome-labelled antibodies using cell permeabilization and fixation reagents (FoxP3/Transcription Factor Staining Buffer Set; eBio- science). Afterwards, cells were stained with surface antibodies (multicolor flow cytometry) specific for CD3/fluorescein isothiocyanate (FITC) (eBioscience), CD4/phycoerythrin (PE) (Miltenyi Biotec, Bergisch Gladbach, Germany), CD25/brilliant violet (BV421; BioLegend, San Diego, CA, USA) followed by intranuclear staining for FoxP3 (eFluor660; eBioscience). FoxP3 staining was conducted according to the manufacturer’s protocol. We used fluorescence activated cell sorter (FACS) staining buffer (eBioscience) to dilute the fixed and stained cells, before storage at 4°C. Flow cytometric analysis was performed within a time-frame of 4 days. We used a BD LSR II cytometer, FACS Diva software (BD Bioscience, San Jose, CA, USA) and FlowJo (Tree Star, Ashland, OR, USA; Version 10.7.1) for Treg analyses. Tregs were determined by their position in the forward-/side-scatter plot (size/granularity) and co-expression of CD3, CD4, CD25 and FoxP3 (**Supplementary Figure S1**). Fluorescence minus one (FMO) controls were used to establish gating boundaries and to identify any background spread of fluorochromes.

### Statistical Analysis

Data analysis was performed using the SPSS data analysis package (Version 26.0; SPSS Inc., Munich, Germany). Differences between groups were evaluated with Fisher’s exact, Mann–Whitney U, Kruskal-Wallis-test and Dunn’s multiple comparison test. A P-value < 0.05 was considered as statistically significant for single tests.

## Results

**Figure 1:**
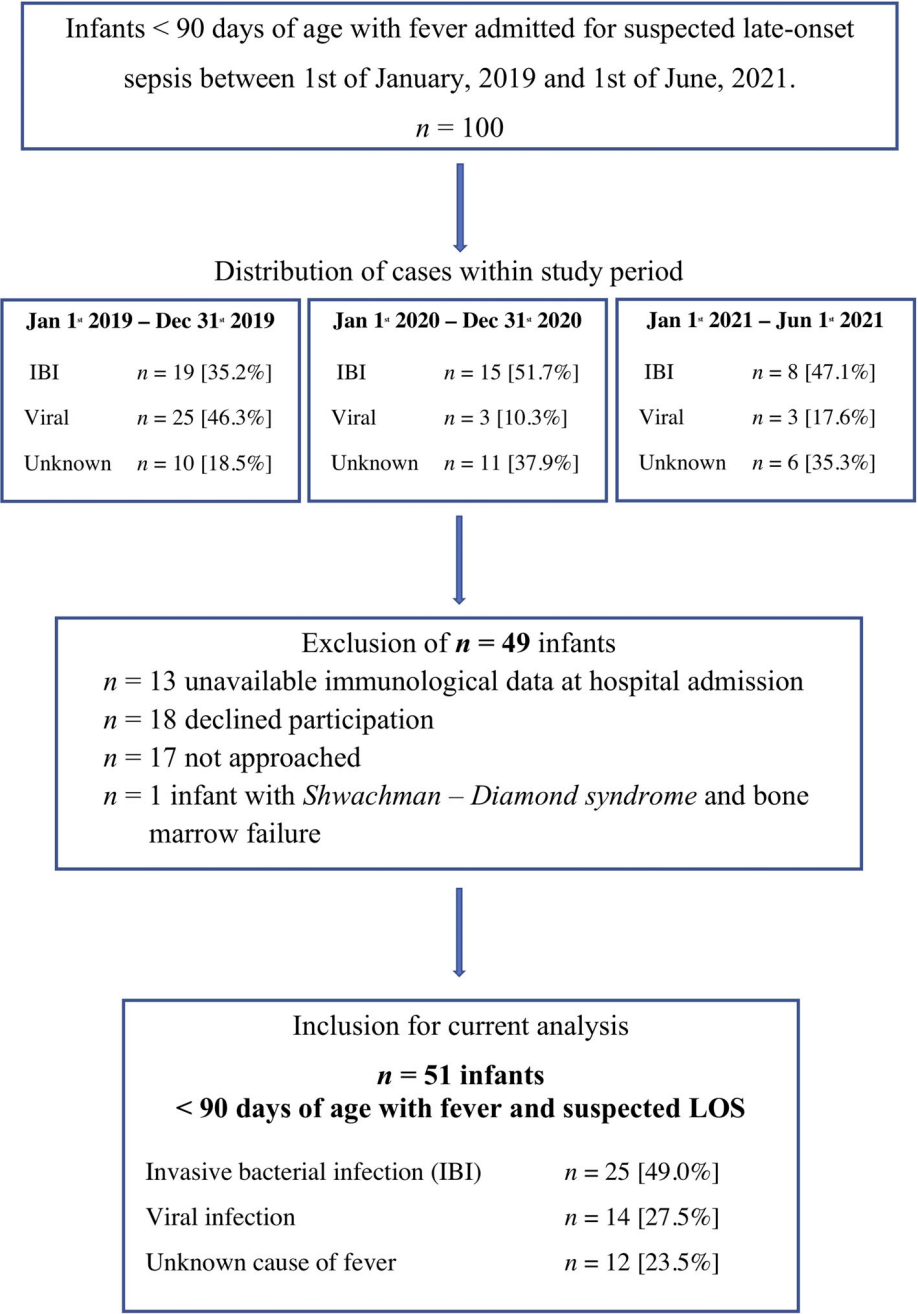
In- and exclusion criteria for the current analysis. IBI, Invasive bacterial infection; Unknown, unknown sourcse of fever; Viral, viral infection. The chronological case distribution demonstrates a significant decline in viral infections that is diagnosed in infants < 90 days of age with suspected LOS in the years 2020 and 2021 that include the SARS Covid-2 pandemic.

During the observational period, we screened all 100 infants with suspected LOS < 90 days of life (**Figure 1**). We excluded 49 infants for the following reasons: lack of immunological data at hospital admission (n=13), denial of consent (n=18), not approached by attending physician (n=17) and underlying Shwachman-Diamond syndrome with bone marrow failure (n=1).

We included *n* = 51 infants < 90 days of age with fever and suspected LOS with a median gestational age of 39.9 weeks (IQR 37.8 – 40.4) weeks, a median birth weight of 3750 grams (IQR 3300g – 4305g) grams and a median age of 45.0 days (IQR 19.0 – 60.0 days) at hospital admission (**Table 1**). The study cohort included 4 former preterm infants > 30 weeks of gestation with uneventful history and 10 infants with postnatal antibiotic treatment due to suspected but unconfirmed early-onset sepsis.

**Table 1:** Clinical characteristics of study cohort.

	Suspected LOS	IBI	Viral infection	No causative pathogen	Control group
**Number** n, (%)	51 (100)	25 (49.0)	14 (27.5)	12 (23.5)	30
**Gestational age** (weeks; median, IQR)	39.9 (37.8 – 40.4)	39.9 (37.3 – 40.4)	39.4 (38.7 – 40.4)	39.9 (37.2 – 40.3)	38.9 (37.3 - 38.8)
**Birth weight** (g; median, IQR)	3750 (3300 – 4305)	4260 (3665 – 5095)	3470 (3250 – 3965)	3573 (2335 – 4118)	4438 (3689 – 5014)
**Gender, male** (%, n)	56.8 (n=29)	64.0 (n=16)	64.3 (n=9)	41.7 (n=4)	73.3 (n=22)
**Delivery mode, vaginal delivery** (%, n)	80.4 (n=40)	68.0 (n=17)	85.7 (n=12)	91.6 (n=11)	56.6 (n=17)
**Age at admission** (days; median, IQR)	45.0 (19.0 – 60.0)	45.0 (15.5 – 61.5)	44.0 (27.0 – 55.5)	41.0 (6.8 – 66.0)	44.5 (18.3 – 61.5)
**Antenatal antibiotics** (%, n)	20.4 (n=15)	40.0 (n=10)	21.4 (n=3)	16.7 (n=2)	6.6 (n=2)
Previous antibiotic exposure* (%, n)	19.6 (n=10)	20.0 (n=5)	14.3 (n=2)	25.0 (n=3)	16.7 (n=6)
**At least one elder sibling** (%, n)	68.6 (n=35)	60.0 (n=15)	85.7 (n=12)	66.7 (n=8)	60.0 (n=12)
**Fever** >38°C/>37.5°C (%, n)	94.1/100 (n=48/51)	92.0/100 (n=23/25)	100/100 (n=14/14)	91.7/100 (n=11/12)	◆
**Length of hospital stay** (d; median, IQR)	7.0 (4.0 – 12.0)	11.0 (8.0 – 14.5)	4.0 (2.8 – 7.0)	5.5. (4.0 – 6.8)	1.5 (1.0 – 3.0)
**Antibiotic therapy** (%, n)	98.0 (n=50)	100 (n=25)	92.9 (n=13)	100 (n=12)	◆
**Duration of antibiotic therapy** (days; median, IQR)	7.0 (4.0 – 10.0)	10.0 (7.0 – 14.0)	3.5 (2.5 – 5.0)	4.5 (4.0 – 6.8)	◆
**Aciclovir** (%, n)	31.4 (n=16)	24.0 (n=6)	21.4 (n=3)	50.0 (n=7)	◆

IBI, invasive bacterial infection; IQR interquartile range; *antibiotic exposure for suspected but unconfirmed early-onset sepsis; ◆ exclusion criteria for controls or not applicable.

The most frequently documented clinical symptoms were fever (body temperature > 38.0°C 94%; > 37.5°C 100%), worsened general condition (78.4%) and agitation (51.0%). Patients were hospitalized for a median of 7.0 days (IQR 4 – 12 days). IBI was diagnosed in 25 (49.0%) infants including two infants with urosepsis and rhinovirus co-infection. 14 infants (27.5%) had proven viral infection (**Table 2**), while no bacterial or viral pathogen was found in 12 infants (23.5%). 85.7% of infants with viral infection had elder siblings, while infants with IBI were often exposed to antenatal antibiotics (40%) and to be delivered by Caesarean section (32%, **Table 1**). Our control group included *n* = 30 infants < 90 days of age that were admitted to hospital for non-infectious reasons: elective surgery for hernia or foot deformity (*n* =15), events that required 24 to 72-hour inpatient monitoring such as apparent life-threatening events (ALTE) or suspected but unconfirmed seizure (*n* = 12), failure to thrive and hyperbilirubinemia (*n* = 3). All infants included in the control group did not have any clinical or laboratory sign of infection or underlying diseases. The cohort included 2 preterm infants of 35 + 1 and 34 + 3 weeks of gestation.

**Table 2:** Bacterial and viral causes of fever in infants <90 days of age.

	Invasive bacterial infection (IBI)		Viral infection	
	n = 25 (49.0%)		n = 14 (27.5%)	
**Focus**	Urosepsis	n = 18	URT infection	n = 6
	BC-positive	n = 9	Meningitis	n = 4
	Meningitis	n = 1	Pneumonia	n = 3
	Endocarditis	n = 1	Gastroenteritis	n = 1
	Pneumonia	n = 2		
**Pathogen**	*E. coli*	n = 15	*Enterovirus*	n = 7
	*GBS*	n = 5	*Rhinovirus*	n = 3
	*Kl. oxytoca*	n = 1	*Rotavirus*	n = 1
	*E. faecalis*	n = 2	*RSV*	n = 1
	*Kl. pneumoniae*	n = 2	*Corona NB*	n = 1
			*Corona NL 63*	n = 1

URT, Upper respiratory tract; BC, blood culture; GBS, group B streptococcus (streptococcus agalactiae); RSV, respiratory syncytial virus; URT infection: virus detected by multiplex PCR from nasopharyngeal aspirate; for n = 12 cases (23.5%) of suspected LOS no microbial pathogen was detected (unknown cause of fever).

### Microbiological Diagnostics

Among infants with IBI, *n* = 5 (20.0%) had *Streptococcus agalactiae (GBS)* culture-proven LOS. Two of these cases were complicated by meningitis, or endocarditis. Two mothers were tested positive for *GBS* during pregnancy, one was tested negative and two mothers had an unknown *GBS*-status. Three infants with *GBS* sepsis had previous ante- and/or postnatal antibiotic treatment for positive maternal *GBS* status and/or suspected early-onset sepsis that was not confirmed retrospectively. Urosepsis caused by *Escherichia coli* was the most frequent cause of IBI, i.e. 15/18 with urosepsis cases. 3 of these infants had a positive blood culture. Other pathogens causing urosepsis were *Klebsiella oxytoca* (*n* = 1) and *Enterococcus faecalis* (*n* = 2). Seven infants with urosepsis were diagnosed with malformation of the urogenital tract (**Table 2**).

### *Enterovirus* Infection Is Frequently Diagnosed in Young Infants With Suspected LOS

Infection with *enterovirus* was the most frequent cause of viral infection resulting in sepsis workups (50% of viral infections). *Enterovirus* meningitis was diagnosed in 4/7 *enterovirus* infections. *Rhinoviruses* were found as causative agent in 3 cases; single cases were caused by *rotavirus*, *respiratory syncytial virus* (*RSV*), *coronavirus NL 63* and *coronavirus NB* infection (**Table 2**). Notably, there was a significant decline in viral infections as a cause of suspected LOS <90 days in the COVID-19 pandemic time-frame of our observational study (**Figure 1**).

### Admission for Sepsis Decodes Underlying Diseases

Interestingly our cohort included 4 infants (7.8%) with suspected or proven major underlying conditions, that were not known before hospital admission such as Trisomy 21, battered – child syndrome (BCS) and two infants with more than one dysmorphic feature and suspected syndrome. Two of these cases were diagnosed with urosepsis and one had pneumonia as potential infectious complication of the underlying disease. In the case of BCS no infectious cause for the symptomatic infant was found.

### Infants <90 Days With IBI Display Lower Frequencies of Regulatory T Cells Than Healthy Controls

**Figure 2:**
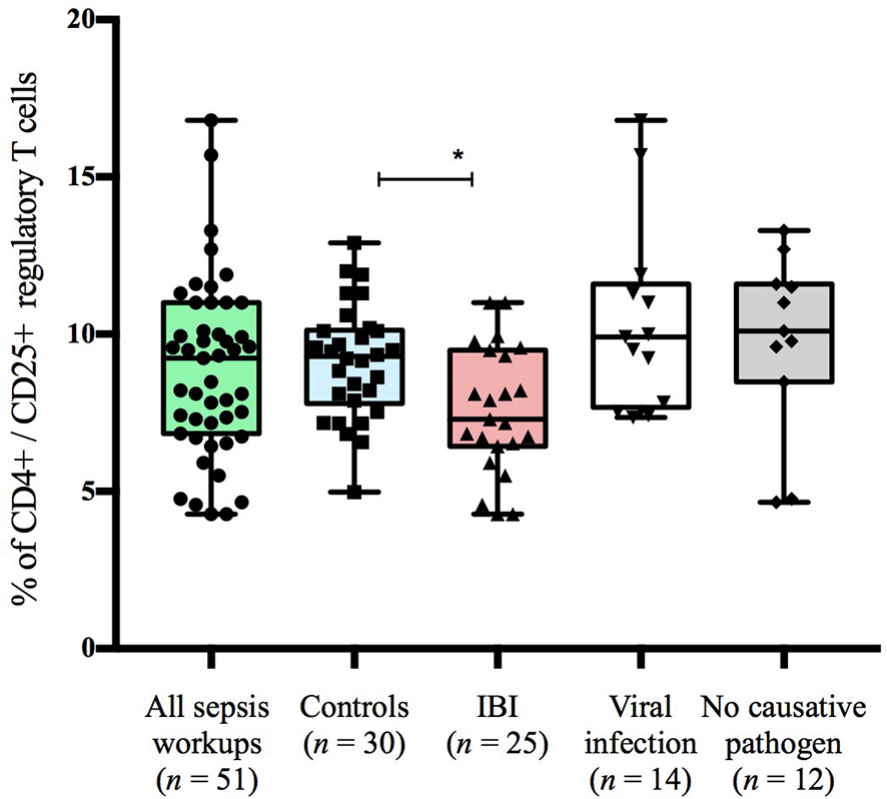
Frequencies of CD4+CD25+ T cells stratified to subgroups of study cohort. Boxes are shown as median and IQR; p-value was derived from Dunn’s multiple comparisons test; *p < 0.01.

**Figure 3:**
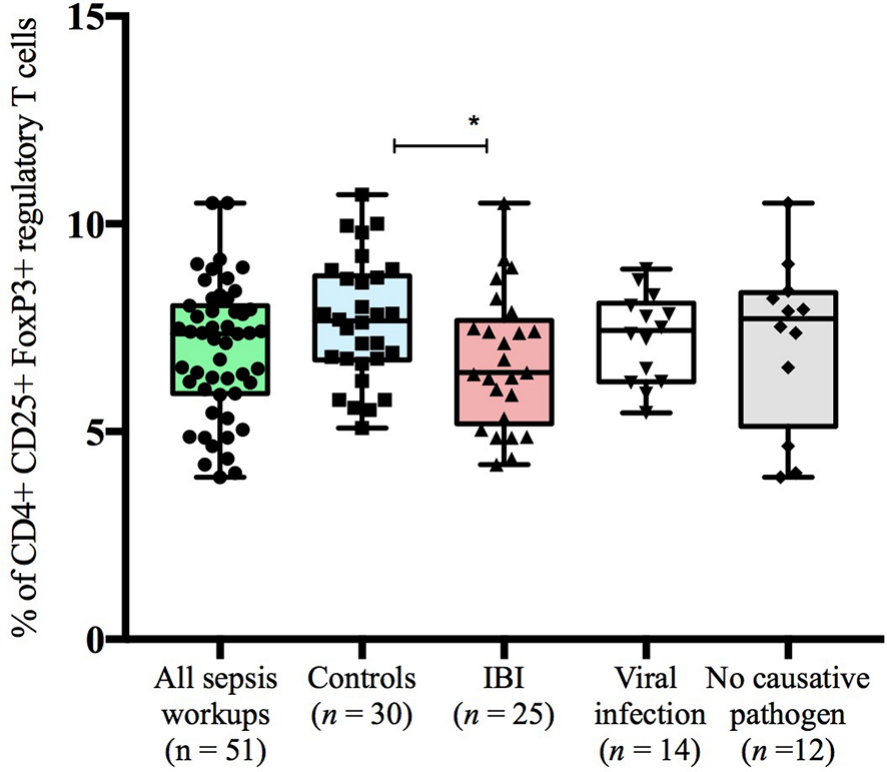
Frequencies of CD4+CD25+FoxP3+regulatory T cells stratified to subgroups of study cohort. Boxes are shown as median and IQR; *p-value was derived from Dunn’s multiple comparisons test (p < 0.05).

**Figure 4:**
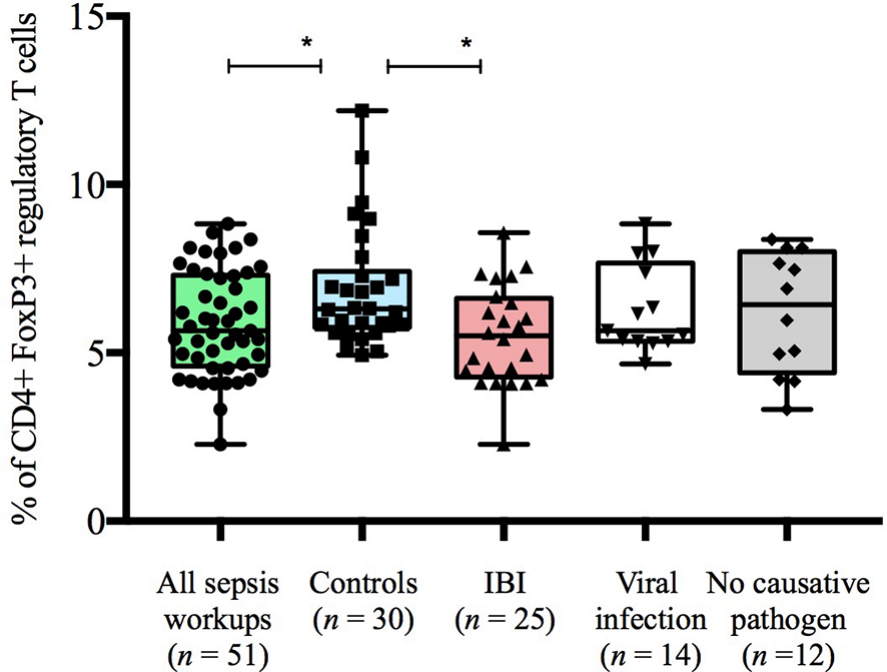
Frequencies of CD4+FoxP3+regulatory T cells stratified to subgroups of study cohort. Boxes are shown as median and IQR; *p-values were derived from Dunn’s multiple comparisons test (p < 0.01).

**Figure 5:**
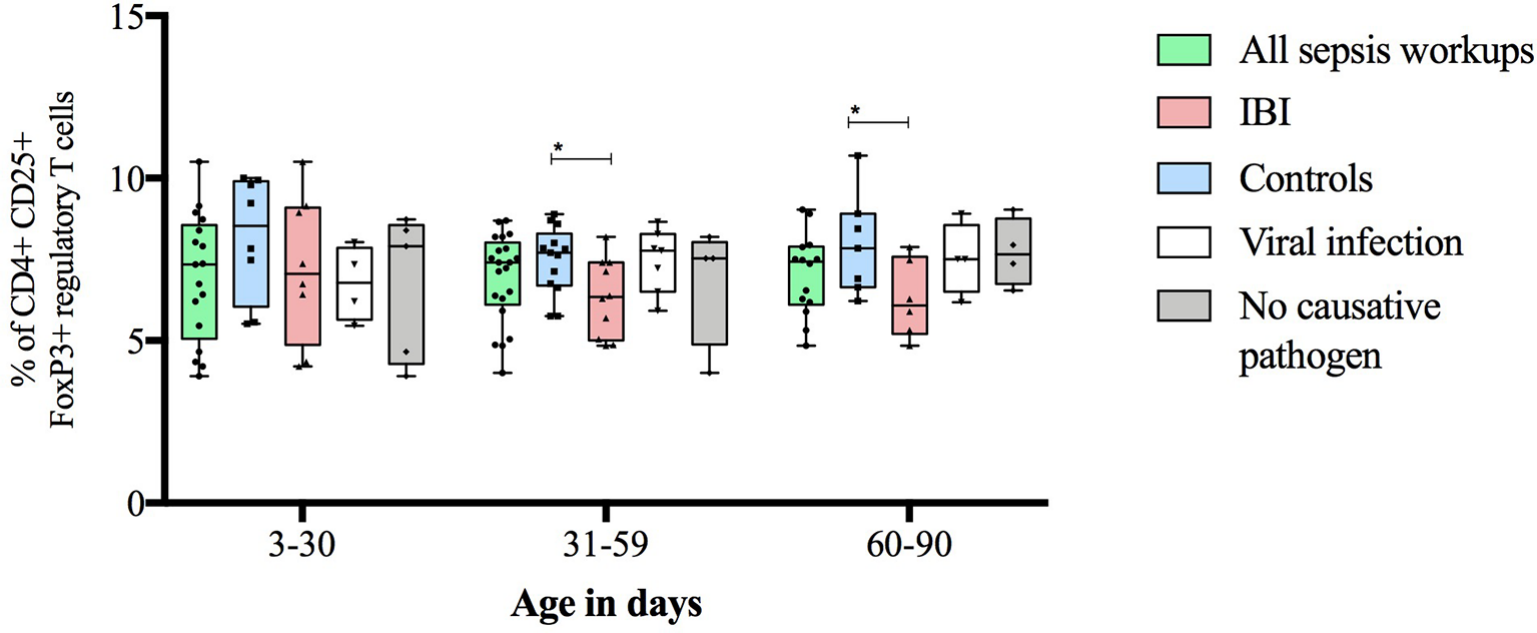
Frequencies of regulatory T cells stratified to infants’ age and subgroups of study cohort. Boxes are shown as median and IQR; whiskers show minimum and maximum values; *p-values were derived from Dunn’s multiple comparisons test (<0.05).

Infants with suspected LOS displayed a decreased abundance of CD4+ FoxP3+ T cells as compared to controls, which was most pronounced in the subgroup of infants with IBI (**Table 3** and **Figures 2**–**4**). In order to account for a potential age-related bias, we divided our cohort in three age-defined subgroups: 3 to 30 days, 31 to 60 days and 61 to 90 days (**Figure 5**). The trend of lower regulatory T cells in infants with IBI was observed in all age groups with significant levels in infants > 30 days of age. Differences with regard to frequencies of CD3+ or CD4+ cells were not observed.

**Table 3:** Frequencies of T cells and Tregs stratified to subgroups.

Markers for Treg detection	All sepsis workups (*n* = 51)	No causative pathogen (*n* = 12)	Viral infection (*n* = 14)	IBI (*n* = 25)	Controls (*n* = 30)
**CD3^+^ (%)**	59.7 [46.1 – 66.1]	58.4 [43.9 – 64.8]	60.4 [44.9 – 66.3]	61.0 [50.3 – 68.0]	59.1 [55.6 – 64.0]
**CD4^+^ (%)**	43.7 [36.1– 51.1]	41.4 [35.8 – 47.5]	47.6 [37.6 – 52.6]	44.7 [33.0 – 54.4]	44.0 [35.8 – 47.0]
**CD4^+^CD25^+^ (%)**	9.5 [7.4 – 11.0]	10.1 [8.5 – 11.6]	9.9 [7.7 – 11.6]	7.5 [6.4 – 9.5]	9.3 [7.8 – 10.1]
				p = 0.009*	
**CD4^+^FoxP3^+^ (%)**	5.6 [4.6 – 7.3]	6.4 [4.4 – 8.0]	5.7 [5.3 – 7.7]	5.4 [4.1 – 6.6]	6.4 [5.8 – 7.4]
	p = 0.009*			p = 0.007*	
**CD4^+^FoxP3^+^CD25^+^Tregs (%)**	7.4 [5.9 – 8.0]	7.7 [6.7 – 8.8]	7.4 [6.2 – 8.1]	6.4 [5.1 – 7.7]	7.7 [6.7 – 8.8]
				p = 0.02*	

Data are expressed by percentages and [IQR]. IBI, invasive bacterial infection; p-values were derived from Dunn’s multiple comparisons test (*) and demonstrate the significance between particular subgroup and control group.

We also noticed elevated HLA-DR-positive CD3+ cells in infants with LOS and a higher CD4/CD8-ratio in infants with viral infection as compared to healthy controls. Infants with viral infections had a higher number of natural killer cells as compared to infants with IBI. No differences were observed in the B-cell and cytotoxic T cell compartment (**Table 4**).

**Table 4:** Lymphocyte subsets and immune cell count of study cohort and subgroups.

Number, n	Suspected LOS	IBI	Viral infection	No causative pathogen	Control group
	n=51	n=25	n=14	n=12	n=22
**White blood cells, cells/nl** (median, IQR)	11.2 (8.3 – 15.5)	12.6 (7.8 – 16.6)	11.1 (8.7 – 14.4)	10.5 (7.9 – 15.1)	10.1 (7.8 – 12.1)
**Lymphocytes, cells/nl** (median, IQR)	5.5 (3.7 – 7.1)	4.4 (3.5 – 6.7)	7.2 (2.8 – 8.2)	6.1 (3.9 – 7.4)	5.7 (4.5 – 7.0)
**Number, n**	**n=45**	**n=23**	**n=14**	**n=8**	**n=22**
**CD3^+^**	3.5 (2.6 – 4.8)	3.5 (2.7 – 4.9)	4.6 (2.9 – 4.9)	2.7 (0.9 – 4.2)	3.3 (2.9 – 4.3)
**CD4^+^**	2.7 (1.9 – 3.9)	2.6 (1.9 – 3.9)	2.9 (2.4 – 3.9)	1.9 (0.6 – 3.2)	2.5 (1.9 – 3.0)
**CD8^+^**	0.8 (0.5 – 1.1)	0.8 (0.6 – 1.0)	0.9 (0.5 – 1.4)	0.7 (0.2 – 1.0)	0.9 (0.6 – 1.0)
**CD19^+^**	1.0 (0.6 – 1.3)	0.8 (0.5 – 1.3)	1.2 (0.7 – 1.4)	0.9 (1.6 – 1.6)	1.0 (0.5 – 1.3)
**(CD16+56) ^+^**	0.4 (0.2 – 0.8)	0.3 (0.2 – 0.6)	0.7 (0.4 – 1.0)	0.5 (0.2 – 0.6)	0.4 (0.3 – 0.6)
		p(V)= 0.01			
**CD4/CD8-ratio**	3.4 (2.6 – 4.5)	3.4 (2.6 – 4.5)	4.0 (3.0 – 4.6)	3.0 (2.6 – 5.7)	2.8 (2.3 – 2.7)
			p(C) = 0.03		
**activated T-lymphocyte**	0.06 (0.03 – 0.1)	0.06 (0.03 – 0.1)	0.08 (0.04 – 0.1)	0.03 (0.03 – 0.08)	0.03 (0.02 – 0.05)
	p(C) = 0.04	p(C) = 0.04	p(C) = 0.03		

IBI, invasive bacterial infection; IQR interquartile range; LOS, late-onset sepsis.

p-values were derived from Dunn’s multiple comparisons test; p(V) p-value when compared to subgroup with viral infection; p(C) p-value when compared to control group.

## Discussion

In this single-centre prospective exploratory study we provide epidemiological and immunological data of young infants < 90 days of age with suspected and proven LOS. IBI occurred in 49.0% of all infants that were admitted for sepsis workup, whereas 27.5% had proven viral infection. Notably, in about one quarter of the cases (23.5%) no infectious cause was found despite thorough phenotyping and extensive microbiological diagnostics. As a specific immunological hallmark, infants with suspected LOS displayed a decreased abundance of CD4+ FoxP3+ T cells as compared to controls, in particular infants with IBI.

It was the main hypothesis of our study that this immunologically unexplored group of infants with suspected community-acquired LOS in the first 90 days of life is characterized by certain immunological specificities. The comprehensive approach of stratification between IBI and viral infection, in this group of patients is crucial for future development of biomarkers that help clinicians’ decision making for initiation and duration of anti-infective treatment. Clinical susceptibility may act in concert with specific, potentially malleable immunological predisposition. Our data suggest that Tregs are diminished in community-acquired IBI in young infants which is a novel observation for sepsis patients. Pagel et al. demonstrated higher Treg frequencies in preterm infants with early-onset sepsis (EOS), independent of gestational age (10). Studies with adult sepsis-patients revealed higher abundances of Tregs during the course of sepsis as well as an association with poor prognosis and increased mortality in patients with septic shock (14). In these studies, the depletion of Tregs was reported to decrease mortality (14, 24). However, the scientific question remains unsolved, whether altered Treg frequencies display a risk factor for the onset of sepsis (as it has been suggested for preterm infants with EOS (10),) or whether the course of sepsis correlates with dysregulation/dysfunction of T cell subsets including regulatory T cells (as it has been proposed for adult patients with septic shock (14),). Animal and human cord blood studies consistently reported essential differences in Treg-associated function and capacity between term and preterm neonates (25–28). Since Tregs play a role for a permissive physiological colonization of young infants (29, 30), we propose that reduced Treg quantities may represent a dysregulation pattern of the mutual immune-microbiome interaction as hallmark of susceptibility to IBI. Additional risk factors may include higher exposure rate to antenatal antibiotics and Caesarean section as noted in the IBI subgroup of our cohort. The dynamics of *GBS* late-onset infections despite positive screening and intrapartum prophylaxis in some IBI infants also suggest fluctuations in host immunity that contribute to failure of natural niche occupation of pathobionts (31). On the other hand, infants with viral disease were most often younger siblings which is a known risk factor for household transmission of viruses. Interestingly, we noted a significant effect of extended hygiene measures during the COVID-19-pandemic, i.e. a decline in cases of suspected LOS that were eventually diagnosed with viral infection.

Decreased quantities of Tregs were found in young infants with *RSV* infection (12). While we only present a trend towards lower Treg frequencies, Raiden et al. studied immunological characteristics in 36 infants with *RSV* infection and described a marked reduction of Treg frequencies from 6.5% to 1% but also less dramatic changes depending on the Treg phenotype (11). An increased recruitment from the infectious focus, i.e. the lungs or lung-draining lymph nodes, higher susceptibility to apoptosis or an increased instability of infant Tregs have been proposed as underlying mechanisms for the Treg reduction in peripheral blood (32, 33).

While CD56^+^ T cells (NK cells) play a key role in the promotion of severe systemic inflammation in adults (34), we found no remarkable differences in NK cell numbers between subgroups of suspected LOS and controls. Infants with viral infections had a higher number of NK cells as compared to infants with IBI which suggest a specific role of NK cells in antiviral immunity even in young infancy (35). We also noticed elevated HLA-DR-positive CD3+ cells (activated T lymphocytes) in infants with LOS and a higher CD4/CD8-ratio in infants with viral infection as compared to healthy controls. Term neonates are known to have higher counts of CD4+ T cells in peripheral blood than adults (36) which is why CD4/CD8 ratio is usually 3:1, and declines to adult values of 2:1 until the age of four years (37). Higher CD4/CD8 ratios as seen in infants with viral infection in our cohort may also be linked with a potential immaturity state carrying increased infection risk.

### Strengths and Limitations

The major strength of our study is the novel approach of providing detailed microbiological, virological and immunological data of a well phenotyped cohort of infants < 90 days of age with suspected sepsis as compared to healthy controls. The topic is of high clinical relevance given the potential long-term consequences of both, failure to treat IBI appropriately and unnecessary exposure to antibiotics early in life. Our study cohort is unique as previous studies on cellular immunity in early life sepsis investigated neonates who had never been discharged home, in particular preterm infants, or assessed healthy babies with regard to developmental immune trajectories (9, 10). There are limitations to our study, i.e. the single center approach, small sample size and lack of pre-sepsis data which would require a large-scale birth cohort study. Our study design was exploratory and based on a convenience sample including differences in available blood volumes for all immunological tests and times from sampling to blood processing. Our study describes quantitative data, whereas functional capacities or single cell signatures are not yet studied in detail and subject to future studies.

## Conclusion

Infants admitted with suspected LOS are characterized by lower quantities of regulatory T cells. Our exploratory data support the concept of a potential immaturity state and failed immune tolerance development for young infants with LOS. Future large-scale studies are needed to elucidate pre-sepsis conditions and to target the microbiome-immunity interplay as a potential risk pattern.

## Data Availability Statement

The data analyzed in this study is subject to the following licenses/restrictions: The datasets generated during and/or analyzed during the current study are available from the corresponding author on reasonable request. Requests to access these datasets should be directed to matsingmar.fortmann@uksh.de.

## Ethics Statement

The studies involving human participants were reviewed and approved by the ethics committee "Ethikkommission der Universität zu Lübeck". Written informed consent to participate in this study was provided by the participants’ legal guardian/next of kin.

## Author Contributions

IF, CH, EH, JR, LT, JL, MD, BS, and JP contributed to conception and design of the study. IF, JP, AH, VVZ and M-TD organized the database. IF performed the statistical analysis. IF and CH wrote the first draft of the manuscript. JP and M-TD wrote sections of the manuscript. All authors contributed to the article and approved the submitted version.

## Funding

The *FWS study* is part of a clinician scientist grant provided by the German Center for Infection Research (DZIF) which is funded by the German Ministry for Education and Research. There has been no involvement in study design, collection of analysis, interpretation of data, writing of the report and decision to submit the manuscript for publication by the German Ministry for Education and Research. The first version of the manuscript was written by IF and CH. No payment, honorarium, grant or other form of payment has been given to the authors. The study was also supported by the University of Lübeck junior research grant (IF), Lübeck-Hilfe für krebskranke Kinder (https://luebeck-hilfe-fuer-krebskranke-kinder.de) and Annemarie-König-Stiftung.

## Conflict of Interest

The authors declare that the research was conducted in the absence of any commercial or financial relationships that could be construed as a potential conflict of interest.

## Publisher’s Note

All claims expressed in this article are solely those of the authors and do not necessarily represent those of their affiliated organizations, or those of the publisher, the editors and the reviewers. Any product that may be evaluated in this article, or claim that may be made by its manufacturer, is not guaranteed or endorsed by the publisher.

## Supplementary Material

Supplementary Figure 1 Gating strategy for flow cytometry analysis of CD3**^+^**, CD4^+^, CD25^+^, forkhead box protein 3 (FoxP3^+^) regulatory T cells. Representative plots for the analyses of different lymphocyte subsets via flow cytometry (10, 23). CD3+ CD4+ cells were gated, a single cell gate was set to exclude doublets and the lymphocytes were determined in the initial steps. Afterwards the lymphocyte gate was backgated on the first population and calculated to a total number of 30.0000 cells in the backgated lymphocyte gate. Total CD3+, CD4+ and CD3+CD4+ cells were determined. The latter were further analyzed and the following subpopulations were gated: CD3+CD4+Foxp3+ Tregs, CD3+CD4+Foxp3+ and CD3+CD4+CD25+ cells. The numbers in the panels indicate the frequency of the gated cells in percentages. FSC, forward-scatter; SSC, side-scatter.

## Abbreviations

FWS, Fever without source; GBS, *group-B-streptococcus (Streptococcus agalactiae)*; IBI, Invasive bacterial infection; GNN, German Neonatal Network; LOS, Late onset sepsis; NPA, Nasopharyngeal aspirate; NICU, Neonatal Intensive Care Unit; Treg, Regulatory T cell; UTI, Urinary tract infection.
