# Supplementary figures and images for: Infants Younger Than 90 Days Admitted for Late-Onset Sepsis Display a Reduced Abundance of Regulatory T Cells
Source: Front Immunol. 2021 Aug 27;12:666447. doi: 10.3389/fimmu.2021.666447 (PMC8430331; doi:10.3389/fimmu.2021.666447)

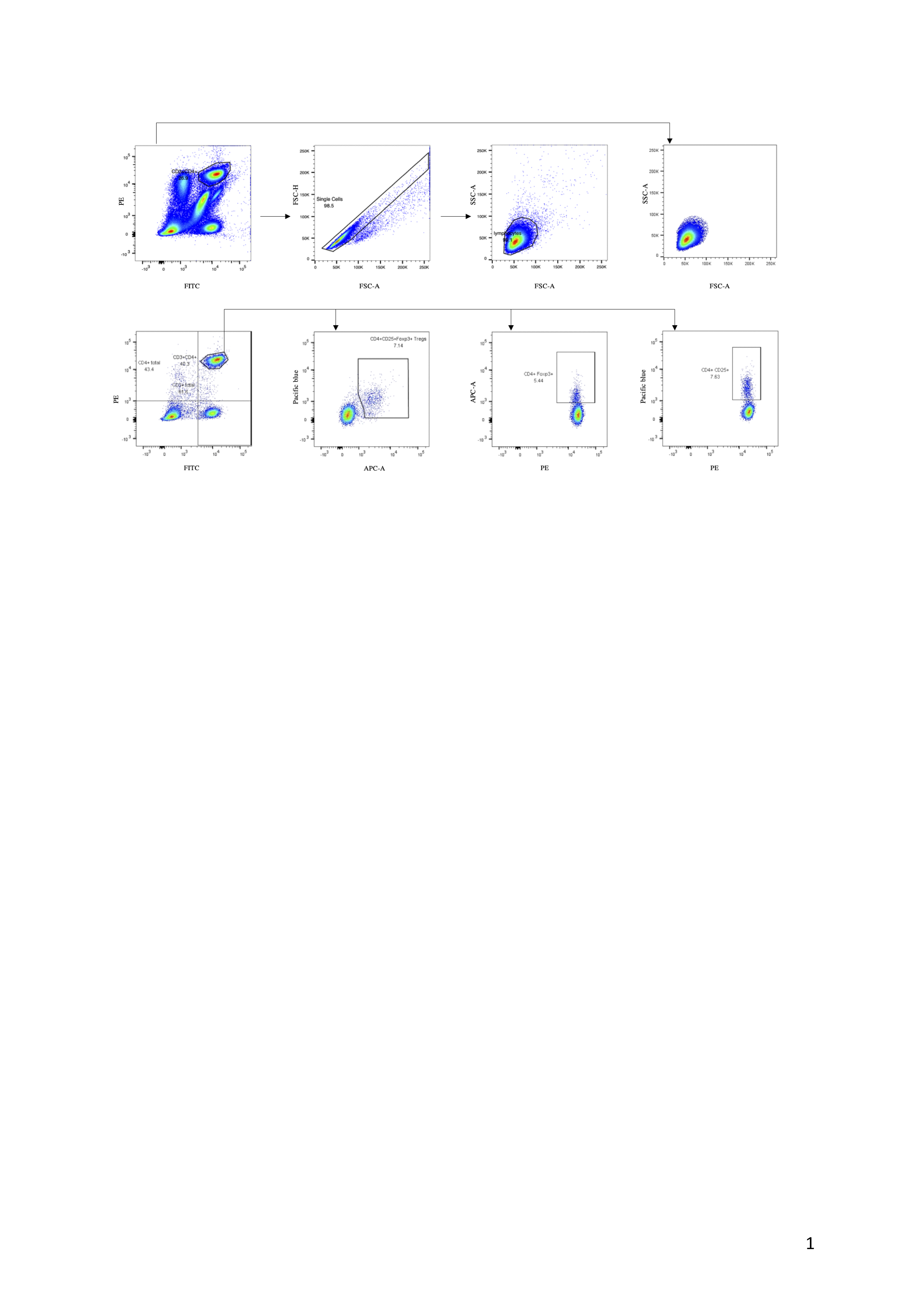

Supplement: Supplementary Figure 1 — Gating strategy for flow cytometry analysis of CD3+, CD4+, CD25+, forkhead box protein 3 (FoxP3+) regulatory T cells. Representative plots for the analyses of different lymphocyte subsets via flow cytometry (10, 23). CD3+ CD4+ cells were gated, a single cell gate was set to exclude doublets and the lymphocytes were determined in the initial steps. Afterwards the lymphocyte gate was backgated on the first population and calculated to a total number of 30.0000 cells in the backgated lymphocyte gate. Total CD3+, CD4+ and CD3+CD4+ cells were determined. The latter were further analyzed and the following subpopulations were gated: CD3+CD4+Foxp3+ Tregs, CD3+CD4+Foxp3+ and CD3+CD4+CD25+ cells. The numbers in the panels indicate the frequency of the gated cells in percentages. FSC, forward-scatter; SSC, side-scatter. [file Image_1.jpeg]
