# Supplementary material for: Infants Younger Than 90 Days Admitted for Late-Onset Sepsis Display a Reduced Abundance of Regulatory T Cells
Source: Front Immunol. 2021 Aug 27;12:666447. doi: 10.3389/fimmu.2021.666447 (PMC8430331; doi:10.3389/fimmu.2021.666447)
Supplement: Supplementary file 2 [file Table_1.docx]

**Suppl. table. S1** Total counts of T cells and Tregs stratified to subgroups

| **Markers for**  **Treg detection** | **All sepsis workups**  (*n* = 51) | **No causative**  **pathogen**  (*n* = 12) | **Viral infection**  (*n* = 14) | **IBI**  (*n* = 25) | **Controls**  (*n* = 30) | **Kruskal-Wallis test** |
| --- | --- | --- | --- | --- | --- | --- |
| **CD3^+^ (total)** | 17912  [13815 - 19813] | 17511  [13159-19458] | 18118  [13455– 19882] | 18292  [15071– 20378] | 17711  [16672- 19199] | p = 0.9 |
| **CD4^+^ (total)** | 12887  [10354 -15151] | 12422  [10722-14246] | 11952  [10035- 14858] | 13404  [9908 – 16334] | 13171  [10689 - 13171] | p = 0.9 |
| **CD4^+^CD25^+^ (total)** | 1203  [908.5 -1328] | 1214  [630 - 1366] | 1288  [928 -1480] | 1153  [889.3 - 1294] | 1212  [927.8 -1330] | p = 0.3 |
| **CD4^+^FoxP3^+^ (total)** | 727  [558 - 941] | 743.5  [395.8 - 1020] | 812  [602 -981] | 671.5  [549.3 - 942.5] | 742  [600.3 - 938.8] | p = 0.9 |
| **CD4^+^FoxP3^+^CD25^+^**  **Tregs (total)** | 900  [643.3 - 1084] | 947  [467 - 1115] | 1010  [651 - 1161] | 792  [655 - 1161] | 922.5  [740.8 - 1089] | p = 0.3 |

***Legend:*** *Data are expressed by median and [IQR]. IBI, invasive bacterial infection; p-values were derived from Kruskal-Wallis test.*
